# Work Adjustments by Types of Occupations Amongst People with Multiple Sclerosis: A Survey Study

**DOI:** 10.1007/s10926-023-10142-2

**Published:** 2023-11-03

**Authors:** Alejandra Machado, Chantelle Murley, Jessica Dervish, Fitsum Sebsibe Teni, Emilie Friberg

**Affiliations:** Division of Insurance Medicine, Department of Clinical Neuroscience, Karolinska Institutet, SE-171 77 Stockholm, Sweden

**Keywords:** Work adjustments, Occupations, Multiple sclerosis, Chronic disease

## Abstract

**Purpose:**

To explore the occurrence of work adjustments for people with multiple sclerosis (MS) across types of occupations (managerial, office, and manual workers).

**Methods:**

All working-aged (20–50 years) residents in Sweden diagnosed with MS were invited to participate in a web-based survey in 2021. Responses were linked to individual-level nationwide registers. Descriptive analyses were conducted to compare sociodemographic and clinical variables across occupations as well as other responses. The odds ratio of having any adjustment at work was determined using multinomial logistic regression.

**Results:**

From all 4412 respondents (52% response rate), 3313 employees were included. The majority were women (72%) and had low (24.2%) or mild disease severity (44.7%). Nevertheless, different work adjustments across occupations were observed. Compared to the other occupations, office workers reported more invisible symptoms, more work adjustments and considered adapted schedules as the most important adjustment. On the contrary, more managers reported having no limiting symptoms and consequently, disclosed their diagnosis less often. They also reported having fewer work adjustments and more opportunities to modify their work than office and manual workers. Manual workers had a higher likelihood to report needing more support at work than office workers and managers. Further, a higher likelihood of having work adjustments was associated with progressive MS, higher MS severity, and invisible symptoms.

**Conclusion:**

A more severe clinical profile of MS was associated with having work adjustments. The physical demands and responsibilities of an occupation play an important role when requesting and getting work adjustments amongst employees with MS.

**Supplementary Information:**

The online version contains supplementary material available at 10.1007/s10926-023-10142-2.

## Introduction

Multiple sclerosis (MS) is the most prevalent chronic inflammatory disease of the central nervous system affecting individuals of working ages [1]. The wide range of symptoms, in addition to the progression of the disease over time, have an important impact on family, social, and work life of people living with MS (PwMS) [2, 3]. Although recent treatments for MS have been shown to ameliorate or slow down the progression of the disease [4, 5], the severity of MS symptoms may hinder PwMS’ work participation [1, 6]. Hence, specific physical and mental health MS limitations confer an increased risk of exiting the workforce as the disease progresses [7, 8], as well as higher use of compensation systems like sickness absence (SA) or disability pension (DP) benefits when compared with the general population [7–10].

Several factors contribute to maintaining the work participation of PwMS’ work participation. Type of occupation has previously been shown to contribute to the level and course of SA and DP amongst PwMS [11], whereby less physically demanding jobs extend the time until DP and facilitate remaining employed [12]. Work adjustments can also be implemented to reduce SA [13] and extend the working life of people with a chronic disease like MS [14]. Moreover, disclosure of MS at work facilitates remaining longer in employment and usually generates an organisational response to provide vocational interventions [15, 16]. Opportunities for such support in the workplace have been shown to be underused by people with chronic diseases [13], suggesting that requesting support at work may be related to other organisational, social, and demographic factors [17].

The negative impact MS may have on work capacity is evident and therefore, interest has grown in the past years on how to facilitate for PwMS to continue working. Specifically, how certain support at work (e.g. empathy in the workplace) and work adjustments (e.g. ergonomic work tools or need for more breaks) enable work participation. Although treatment advances and work adjustments have extended the working lives of PwMS, knowledge is lacking on the occurrence of support at work and work adjustments (hereafter collectively termed work adjustments), as well as the perceived importance of different types of work adjustments (based on factors like the job’s physical demand or responsibility over others), their MS severity or perceived limiting symptoms. Therefore, we aimed to explore if employed PwMS have work adjustments and what type of adjustments they consider most important to facilitate their work, in general, and across types of occupations, as well as other associated sociodemographic, workplace and clinical factors.

Moreover, we aimed to investigate how PwMS manage their MS symptoms in the workplace and how it differs across occupations, namely if they disclose their diagnosis, require support at work, and whether they can influence their work conditions.

## Methods

A cross-sectional study of working-aged PwMS in Sweden was conducted, utiliszing web-based survey information and national register data.

### Study Population

All individuals aged 20–50 years old, living in Sweden and listed in the Swedish MS Registry (SMSreg) [18, 19] were invited to answer a web-based questionnaire in 2021. Of a total of 8458 adult PwMS invited to participate, 4412 (52%) answered the survey after four reminders. Of these, 3313 (75.1%) were categorised as being “employed” after answering “employed” as their current occupation and not simultaneously reporting “no current occupation” or studying, job-seeking or unable to identify occupation.

### Data Sources

The survey data, administered by Statistics Sweden [20], were linked to individual-level data from nationwide registers. The pseudo-anonymised data was later delivered to the researchers. The Longitudinal Integration Database for Health Insurance and Labour Market Studies (LISA) [21] held by Statistics Sweden, was used to obtain socio-demographic variables (sex, educational level, country of birth, civil status, living with children at home, and type of living area). The following clinical variables were obtained from the SMSreg:

- Type of MS (relapsing–remitting, secondary-progressive, primary-progressive, missing)
- MS severity was assessed with the most recently available Expanded Disability Status Scale score (EDSS). EDSS scores occurring within three years of the survey response were included (n = 323 set to missing) and participants with secondary-progressive or primary-progressive MS with an EDSS score of 0 were set to missing (n = 12). The scores were then categorised (low (EDSS = 0), mild (1–2.5), moderate (3–5.5), and severe (6–9.5) or missing). Moderate and severe scores were collapsed together due to small sample sizes.
- Ongoing treatment (yes, no)

Finally, both years since diagnosis and age were calculated based on the date of survey response.

From a survey comprising 66 questions on work, health, and life-balance as well as the impact of MS on these dimensions, questions on work adjustments (3 questions), type of occupation (1), most limiting symptom (1) and disclosure to the employer (1) were utilised. The translations of the responses to open-ended questions from Swedish to English were examined by the research team to be as close as possible to the original formulation.

### Outcomes

#### Types of Occupations

Occupational groups were classified based on responses to the open question “*What is your current occupation?*”. The answers were originally grouped using the Swedish Standard Classification of Occupations (SSYK 2012) [22] as a reference. This SSYK-2012 is based on the International Labour Organisation’s (ILO) Standard Classification of Occupation 2008 (ISCO–08) [23]. Further, and for the purpose of this study, these groups were then recategoried into: *managers* or *responsible for personnel* (occupations reflecting leadership and/or management of staff or administration of a group combined with no or very low physical demands); *office or low physical demands* (occupations involving lower physical activity demands, e.g. administration, education, health, technology, social work, culture); or *manual or high physical demands* (occupations involving high physical activity, e.g. construction and manufacturing, cleaning or domestic service, industrial work, occupations in machine manufacturing and transport, reparation services, sailors, dock and ramp staff, and recycling station workers).

#### Work Adjustments

The following question inquired about the prevalence of work adjustments: *“Do you have any adjustments that helps you with your work?”.* Participants could select one of the responses: (1) Yes; (2) Yes, but not enough; (3) No, but I do need; (4) No, not needed. The responses were collapsed for posterior statistical purposes into Yes or No.

#### Type of Work Adjustments that Facilitate Work

The most important type of work adjustment was based on the responses to the open question “*What are the three most important adjustments that facilitate or could facilitate your work?*”. The first response was selected and categorised using the work adjustments framework from the Swedish Work Environment Agency (AFS 2020:5) [24]: (1) Special work equipment or environment; (2) Customised tasks; (3) Flexible and reduced working hours (hereafter, adapted working schedule); and (4) Other support (change division of labour or other responses that did not meet the previous descriptions). Finally, responses containing “did not know” or left blank were assigned to (5) not applicable (N/A).

#### Opportunity to Influence Work

The participants’ opportunity to influence or adjust certain work conditions was assessed by asking: *“In your work, to what extent do you have the opportunity to… a) influence your working hours? b) work remotely /from home when you need to? c) modify certain work tasks? d) postpone certain work tasks? e) get help? f) take a break when you need to?”. *Participants were able to respond: (1) Always; (2) Often; (3) Sometimes; (4) Rarely; and (5) Never. Positive responses (i.e. always, often, and sometimes) were collapsed for statistical purposes.

#### Disclosure

Regarding disclosure of MS at work, participants were asked the following question: *“Have you disclosed your MS diagnosis to your closest boss?”* where answers were selected from the following options: (1) Yes; (2) No, but I will; (3) No; or (4) Not applicable (when the question might not fit their working circumstances (e.g. being the boss oneself). The no and partly no responses were collapsed for statistical purposes.

#### The most Limiting Symptom

The responses to the open-ended question “*Which MS symptom do you experience as the most limiting?*” informed on the most limiting symptom*.* The responses were grouped into categories using the framework of symptom management from the UK MS Clinical Guidelines [25], and the American National MS Society [28], and discussed amongst the authors, as reported elsewhere [26]. Accordingly, the most limiting symptom responses were categorised into nine groups and reclassified into three groups for statistical purposes (Table1).

**Table 1:** Classification of the most limiting symptom

9 categories	3 categories
No limiting symptom	No limiting symptom
Fatigue	Invisible symptoms
Sensory and pain symptoms	
Mood problems/depression	
Others^a^	
Movement/ambulation problems	Visible symptoms
Vision problems	
Bladder, bowel, and sexual dysfunction	
Dysphonia, dysarthria, and dysphagia	

Adapted from Teni et al. 2023 [26]

^a^Medication-related symptoms, unspecified symptoms, or other psychosocial implications of MS symptoms

### Data Analyses

Descriptive statistics were used to summarise all demographic, clinical, and work-related characteristics of the study population. Responses to the survey questions were also summarised for the total study population and by type of occupation. Differences across the occupations in proportions responding to the survey were assessed with chi-square tests if a categorical variable or ANOVAs for the continuous variables. Missing values were excluded from the comparisons. All multiple comparisons were adjusted with Benjamini–Hochberg procedure.

Multinomial logistic regression analyses assessed the predictors for work adjustments. A stepwise backward method was used to select the covariates when adjusting for demographic and clinical characteristics. Crude and adjusted odds ratios (OR) with 95% confidence intervals (CI) were used to present the results. Missing value categories were omitted in the analyses.

Statistical analyses were performed using SPSS 27 and a p-value of < 0.05 was considered statistically significant.

The participants’ responses to the open-ended questions were analysed using inductive content analysis [27]. NVivo v.11 [28] supported the dataset organisation and the coding of the responses. A researcher (JD), with training in qualitative methods and analysis, carefully examined and iteratively coded the responses.

## Results

The average age amongst the 3313 employees with MS participating in this study was 40 (Sd = 6.9) and an average of 8.7 years (Sd = 6.7; n = 3068) since their MS diagnosis. The participants were predominantly: women (72.0%); born in Sweden (89.8%); had relapsing–remitting MS (93.9%); and a low (EDSS = 0; 24.2%) or mild disease severity (EDSS = 1–2.5; 44.7%).

Nearly all participants had permanent employment (90.9%). The participants were distributed across occupations: office (79.9%), managers (13.3%), and manual workers (6.9%). Women were mostly represented in the office and managers categories. The office workers also had higher proportions of participants with moderate or severe MS severity compared with the other occupational groups (23.3%). Managers were on average older. The manual group was comprised of men to a larger extent. Manual workers were also younger and had higher proportions without university education (81.1%). They also had a shorter time on average since their diagnosis (7.2 years). Further characteristics are shown in Table 2.

**Table 2:** Descriptive characteristics of the total study population and stratified and compared across types of occupations

		Types of occupations			Total
		Managers	Office	Manual	
		(n = 442)	(n = 2644)	(n = 227)	(n = 3133)
		n (%)	n (%)	n (%)	n (%)
Sociodemographic characteristics					
Sex	Women^a,b,c^	281 (63.6)	2018 (76.3)	85 (37.4)	2384 (72.0)
	Men	161 (36.4)	626 (23.7)	142 (62.6)	929 (28.0)
Age (years)	20–29^a,b^	21 (4.8)	244 (9.2)	26 (11.5)	291 (8.8)
	30–39^a,b,c^	117 (26.5)	880 (33.3)	92 (40.5)	1089 (32.9)
	40–49^a,b,c^	271 (61.3)	1379 (52.2)	100 (44.1)	1750 (52.8)
	50+^d^	33 (7.5)	141 (5.3)	9 (4)	183 (5.5)
	Mean (Sd)^a,b^	41.7 (0.3)	40 (0.1)	38.7 (0.5)	40.1 (6.9)
	(Range)	[21–51]	[20–51]	[20–51]	[20–51]
University level education	Yes^b,c^	303 (68.6)	1813 (68.6)	43 (18.9)	2159 (65.3)
	No	138 (31.2)	827 (31.3)	184 (81.1)	1149 (34.7)
Born in Sweden	Yes	400 (90.5)	2364 (89.4)	211 (93)	2975 (89.8)
	No	42 (9.5)	280 (10.6)	16 (7)	338 (10.2)
Civil status	Married/cohabitant^a,b,c^	201 (45.5)	1440 (54.5)	145 (63.9)	1786 (53.9)
	Single/divorced/widowed	241 (54.5)	1204 (45.5)	82 (36.1)	1527 (46.1)
Living with children	Yes^b,c^	282 (63.8)	1569 (59.3)	114 (50.2)	1965 (62.7)
	No	160 (36.2)	1075 (40.7)	113 (49.8)	1394 (37.3)
Type of living area	Cities^b,c^	208 (47.1)	1217 (46)	66 (29.1)	1487 (44.9)
	Towns and suburbs	175 (39.6)	1001 (37.9)	99 (43.6)	1275 (38.5)
	Rural areas^b,c^	59 (13.3)	426 (16.1)	62 (27.3)	547 (16.5)
Clinical characteristics					
Years since MS diagnosis	0–4^b,c^	130 (29.4)	712 (26.9)	90 (39.6)	932 (28.1)
	5–9	113 (25.6)	714 (27.0)	57 (25.1)	884 (26.7)
	10–14^c^	82 (18.6)	568 (21.5)	31 (13.7)	681 (20.6)
	15+	87 (19.7)	456 (17.2)	28 (12.3)	571 (17.2)
	Missing	30 (6.8)	194 (7.3)	21 (9.3)	245 (7.4)
	Mean (Sd)^b,c^	8.8 (0.3)	8.8 (0.1)	7.2 (0.4)	8.7 (0.1)
	(Range)	[0–29]	[0–33]	[0–25]	[0–33]
Type of MS	RRMS	422 (95.5)	2473 (93.5)	217 (95.6)	3112 (93.9)
	Progressive MS (PP & SP)	15 (3.4)	148 (5.6)	9 (4.0)	172 (5.1)
	Missing	5 (1.1)	23 (0.9)	1 (0.4)	29 (0.9)
MS severity^e^	Low (EDSS = 0)	124 (28.1)	624 (23.6)	54 (23.8)	802 (53.9)
	Mild (EDSS = 1–2.5)	207 (46.8)	1199 (45.3)	82 (36.1)	1488 (44.9)
	Moderate-severe (EDSS = 3–9.5)^a^	37 (8.4)	335 (23.6)	21 (9.3)	393 (11.8)
	Missing	74 (16.7)	486 (28.4)	70 (30.8)	630 (19.0)
Ongoing MS treatment	Yes	407 (92.1)	2419 (91.5)	213 (93.8)	3039 (91.7)
	No	35 (7.9)	225 (8.5)	14 (6.2)	274 (8.3)

Differences between types of occupations (p < .05) are based on two-sided tests and adjusted for all pairwise comparisons using the Benjamini–Hochberg correction or Bonferroni in case of comparison of continuous variables

*n* sample size, *Sd* Standard deviation, *RRMS* Relapsing–Remitting Multiple Sclerosis, *PP* Primary Progressive, *SP* Secondary Progressive, *EDSS* Expanded Disability Status Scale

^a^Proportional difference between Managers and Office workers (p < 0.05)

^b^Proportional difference between Managers and Manual workers (p < 0.05)

^c^Proportional difference between Office and Manual workers (p < 0.05)

^d^PwMS with 50–51 years of age (the survey was sent out for those with a maximum of 50 years old, but they could have turned 51 by the time they completed the survey)

^e^Moderate [EDSS = 3–5.5, n (%) = 328(12.7)] and severe [EDSS = 6–9.5, n (%) = 65(1.9)] have been collapsed into Moderate-severe [EDSS = 3–9.5, n (%) = 293(14.6)] due to the small sample size of the latter group

The survey responses are presented in Table 3.

**Table 3:** Frequencies and proportion of responses from the selected survey questions for the total study population and stratified and compared across types of occupations

	Types of occupations			Total (n = 3133)
	Managerial (n = 442)	Office (n = 2644)	Manual (n = 227)	
	n (%)	n (%)	n (%)	n (%)
Most reported limiting symptom				
No limiting symptom^a,b^	78 (17.7)	341 (12.9)	29 (12.8)	448 (13.5)
Visible symptoms	80 (18.1)	417 (15.8)	37 (16.3)	534 (16.1)
Invisible symptoms^a^	251 (56.8)	1690 (63.9)	130 (57.3)	2071 (62.5)
Missing^b,c^	33 (7.5)	196 (7.4)	31 (13.7)	260 (7.8)
Do you have any adjustments that helps you with your work?				
Yes^a^	42 (9.5)	425 (16.2)	34 (15.0)	501(15.1)
Yes, but not enough	21 (4.7)	135 (5.1)	16 (7.1)	172(5.2)
No, but I do need	32 (7.3)	269 (10.2)	24 (10.6)	325(9.8)
No, not needed^b,c^	346 (78.5)	1802 (68.5)	153 (67.4)	2301(69.5)
Missing	1 (0.2)	11 (0.4)	2 (0.9)	14(0.4)
If you consider these adjustments that facilitate or those that could facilitate your work, which is the most important? (First response)				
N/A^a,b^	354 (80.1)	1887 (71.4)	163 (71.8)	2404 (72.6)
Special equipment/environment	42 (9.5)	350 (13.2)	29 (12.8)	421 (12.7)
Customised tasks	19 (4.3)	105 (4.0)	13 (5.7)	137 (4.1)
Adapted working schedule^a^	23 (5.2)	255 (9.6)	15 (6.6)	293 (8.8)
Other support	4 (0.9)	47 (1.8)	7 (3.1)	58 (1.8)
In your work, to what extent have you had the opportunity to…^d^				
Influence your working hours?^a,b,c^	368 (83.3)	1933 (73.1)	126 (55.5)	2427 (73.3)
Work remotely/from home when you need to?^a,b,c^	359 (81.2)	1485 (56.2)	32 (14.1)	1876 (56.6)
Modify certain work tasks?^a,b,c^	335 (75.8)	1696 (64.1)	110 (48.5)	2146 (64.8)
Postpone certain work tasks?^a,b,c^	313 (70.8)	1535 (58.1)	106 (46.7)	1954 (59.0)
Get help?	228 (51.6)	1410 (53.3)	114 (50.2)	1752 (52.9)
Take a break when you need to?^a,b^	372 (84.2)	2046 (77.4)	174 (76.7)	2592 (78.2)

Differences between occupations (p < .005) are based on two-sided tests and adjusted for all pairwise comparisons using the Benjamini–Hochberg correction

^a^Proportional difference between Managers and Office (p < 0.05)

^b^Proportional difference between Managers and Manual (p < 0.05)

^c^Proportional difference between Office and Manual (p < 0.05)

^d^The n (%) of every statement corresponds to the collapsed positive responses (“always”, “often” and, “sometimes”) for each occupational group

Overall, 62.5% of employees with MS reported having invisible symptoms as their *most limiting symptom***.** Office workers reported invisible limiting symptoms (63.9%) to a significantly higher extent than managers (56.8%). In contrast, a higher percentage of managers reported no limiting symptom (17.7%) than office (12.9%) or manual (12.8%) workers. Complementary analysis also confirmed differences in reporting more invisible and visible limiting symptoms when in mild, moderate, and severe levels of disability (EDSS > 0) than those reporting low disability (EDSS = 0; data not shown).

The majority of employed PwMS (79.0%) had *disclosed their MS to their employer* whilst the rest had not disclosed (17.0%), were considering it (“No, but will do”; 1.8%) or reported “not applicable” (2.2%). Disclosure amongst managers was significantly less frequent compared to office and manual workers (data not shown).

When enquiring about the *adjustments that facilitate or could facilitate their work*, more than two-thirds of the participants responded that they did not need work adjustments (69.5%, n = 3313), 15.1% answered they had some adjustments, and 5.2% reported they had adjustments but not sufficient. Finally, some participants reported not having adjustments but considered they needed adjustments (9.8%). Work adjustments (based on the “yes” answer) were significantly more common amongst office workers compared to managers (Table 3). Importantly, from all respondents who reported having work adjustments (“yes” + “yes, but not enough”; 20.3%, n = 672), 95.5% of them reported having disclosed their MS to their employer (data not shown).

The *types of adjustments* are summarised in Table 3. Only the first response was presented as similar results were shown between percentages of these categories in all three responses. Most participants did not answer this question (N/A, 72.6%), and the majority of them (96.3%) previously answered not needing any support or adjustment at work (see Supplementary material, Table S1). However, some significant differences between occupations were present. Office workers described that adapting schedules facilitated their work to a higher extent (9.6%) compared to managers (5.2%). Whereas managers showed significantly higher proportions (80.1%) of no-response or not applicable (N/A) compared to office (71.4%) or manual workers (71.8%; Table 3). When exploring only the types of adjustments of those that did report having work adjustments (20.3%), the overall categorised responses were: special equipment or environment (45.2%), adapted working schedule (27.6%), customised tasks (12.3%) or other support (5.6%), with no differences across types of occupations (χ2_(8)_ = 12.436; p = 0.133).

Finally, when asked about the *opportunity to influence certain work conditions*, more than half of the participants responded positively (collapsed answers of “always”, “often”, and “sometimes” responses) to every statement. Differences were observed across the occupations, showing a descendent gradient in the rates between managers, office, and manual workers in almost all statements (i.e. “Influence their working hours”, “Work remotely when needed”; “Modify certain work tasks”; “Postpone certain tasks” and “Take a break when needed”). No differences were found across occupations when inquiring about the opportunity to “Get help”.

The results of the multinomial logistic regression analyses are presented in Table 4. The unadjusted models are available in the supplementary material (Table S2). In all adjusted models (Table 4), lower ORs of reporting adjustment at work were observed amongst men as well as amongst younger age groups. Moreover, when predicting adjustment at work only with sociodemographic variables (model 1), PwMS with lower educational level, not being born in Sweden, not living with children, and living outside cities, had higher likelihood of reporting having some support at work (“Yes” and/or “yes but not enough”) or in need of support (“No but I do need”). Further, managers were less likely to report having support at work compared to office workers. When clinical factors (model 2) and other survey responses were included (model 3), greater odds of reporting having or needing any adjustments at work were associated with a progressive type of MS and higher EDSS scores. Further, higher ORs for adjustment at work were also associated with reporting invisible symptoms as the most limiting symptom. As expected, employees with MS had lower ORs to report having any adjustment at work when reporting no limiting symptom or not having disclosed their diagnosis to their employer.

**Table 4:** Odds ratios (OR) and 95% confidence interval (CI) for responses of “having any adjustment that helps with their work” by sociodemographic and clinical variables, as well as responses to other survey questions

	Adjusted model 1			Adjusted model 2			Adjusted model 3		
	OR (95% CI)			OR (95% CI)			OR (95% CI)		
	“Yes”	“Yes, but not enough”	“No, but I do need”	“Yes”	“Yes, but not enough”	“No, but I do need”	“Yes”	“Yes, but not enough”	“No, but I do need”
Sex (ref. = Women)									
Men	**0.58 (0.46–0.74)**	**0.48 (0.32–0.72)**	**0.45 (0.33–0.61)**	**0.55 (0.42–0.72)**	**0.48 (0.30–0.74)**	**0.48 (0.34–0.67)**	**0.64 (0.47–0.85)**	**0.53 (0.33–0.86)**	**0.51 (0.36–0.73)**
Age, years (ref. = 40–49)									
20–29	**0.35 (0.22–0.55)**	**0.49 (0.25–0.96)**	1.10 (0.73–1.65)	**0.59 (0.36–0.97)**	0.72 (0.33–1.59)	1.44 (0.91–2.28)	**0.50 (0.29–0.85)**	0.68 (0.30–1.52)	1.31 (0.81–2.10)
30–39	**0.60 (0.47–0.75)**	0.79 (0.55–1.12)	0.95 (0.73–1.24)	**0.75 (0.58–0.97)**	0.93 (0.61–1.40)	1.04 (0.77–1.41)	**0.68 (0.52–0.90)**	0.88 (0.58–1.34)	1.00 (0.73–1.37)
50+	1.15 (0.78–1.69)	1.14 (0.60–2.15)	**0.45 (0.22–0.95)**	1.16 (0.74–1.83)	0.89 (0.42–1.91)	0.44 (0.18–1.03)	1.08 (0.66–1.76)	0.91 (0.42–1.99)	0.50 (0.21–1.20)
Educational level (ref. = University)									
Non-university	**1.55 (1.25–1.92)**	1.35 (0.95–1.90)	**1.32 (1.02–1.71)**						
Country of birth (ref. = Sweden)									
Not Sweden	1.38 (0.99–1.92)	**1.72 (1.08–2.73)**	**1.52 (1.06–2.18)**						
Living with children (ref. = yes)									
No	**1.45 (1.18–1.79)**	**1.39 (1.00–1.94)**	1.20 (0.93–1.55)	**1.29 (1.02–1.64)**	1.36 (0.93–1.98)	1.18 (0.88–1.58)	**1.43 (1.11–1.85)**	1.47 (1.00–2.17)	1.24 (0.91–1.67)
Type of living area (ref. = Cities)									
Towns and suburbs	**1.34 (1.07–1.68)**	0.98 (0.69–1.40)	1.05 (0.81–1.36)						
Rural areas	**1.53 (1.16–2.02)**	1.13 (0.73–1.77)	0.86 (0.59–1.23)						
Type of occupation (ref. = Office)									
Managerial	**0.52 (0.37–0.73)**	0.85 (0.53–1.37)	0.69 (0.47–1.02)				**0.65 (0.44–0.97)**	1.04 (0.59–1.83)	0.86 (0.56–1.34)
Manual	0.92 (0.61–1.39)	1.61 (0.89–2.89)	1.25 (0.77–2.03)				1.16 (0.67–2.00)	**2.18 (1.07–4.45)**	1.51 (0.83–2.76)
MS type^a,b^ (ref. = RRMS)									
Progressive MS (PPMS & SPMS)				**2.02 (1.27–3.23)**	**2.18 (1.15–4.14)**	1.01 (0.49–2.11)	**2.03 (1.24–3.33)**	**2.46 (1.27–4.78)**	0.90 (0.41–1.99)
MS severity^b^ (ref. = EDSS = 1–2.5)									
EDSS = 0				**0.39 (0.29–0.54)**	**0.25 (0.13–0.46)**	**0.48 (0.35–0.67)**	**0.47 (0.33–0.66)**	**0.29 (0.15–0.56)**	**0.60 (0.42–0.85)**
EDSS = 3–9.5				**3.55 (2.62–4.80)**	**3.92 (2.52–6.10)**	**2.41 (1.63–3.56)**	**2.90 (2.10–4.00)**	**3.50 (2.22–5.51)**	**2.12 (1.41–3.17)**
Most limiting symptom^b^ (ref. = Visible symptoms)									
Invisible symptom							**1.50 (1.10–2.07)**	**2.30 (1.37–3.86)**	**1.81 (1.22–2.68)**
No limiting symptom							**0.35 (0.18–0.66)**	0.23 (0.05–1.04)	**0.10 (0.03–0.34)**
Disclosure^a,b^ (ref. = Yes)									
No							**0.15 (0.09–0.26)**	**0.36 (0.18–0.69)**	0.88 (0.62–1.25)

Reference group (outcome): "No, do not need any support"

Bold numbers indicate a p-value < 0.05, a two-sided test

*ref*. references, *RRMS* Relapsing–remitting MS, *PPMS* Primary progressive MS, *SPMS* secondary progressive MS, *EDSS* Expanded Disability Status Scale

^a^Variables collapsed for analysis purposes. Progressive MS contains PPMS and SPMS. Disclosure “No” contains “No”, “No, but will do” and “No, not needed” responses

^b^Missing observations were not included in the model

## Discussion

This study explored experiences and perspectives of PwMS in paid work regarding work adjustments, their perceptions of the most important type of adjustments, and the possibility of influencing work conditions across different occupations. Additionally, their most limiting symptom and disclosure of MS diagnosis in the workplace were investigated together in association with having support/adjustment at work, highlighting the importance of disclosing MS to access work adjustments.

Overall, our findings show that eight of every ten PwMS in paid work reported having a limiting symptom from their MS, and most of them had disclosed their diagnosis at work. However, only two of every ten reported having work adjustments. In fact, a majority reported not needing support to facilitate their work and more than half of the employed PwMS reported having the possibility to influence or modify their work. Differences were present in most of the inquired topics across the three types of occupations. Office workers reported higher rates of invisible symptoms and considered an adapted working schedule as the most important adjustment at work. On the contrary, PwMS in managerial positions reported having no limiting symptoms to a higher extent. Managers also reported having fewer adjustments at work, disclosed their MS diagnosis to their employer to a lesser extent, and had greater opportunities to influence or modify conditions of their work(place) compared to office and manual workers. On the other hand, manual workers reportedly disclosed their diagnosis to their employer to a higher extent and had lower opportunities to influence or modify the conditions of their work(place) compared to managers and office workers. Accordingly, the type of occupation and the ability to influence work are important factors for facilitating work amongst PwMS.

The rate of disclosing the diagnosis at work within our study was equivalent to a recent systematic review where 60–90% of workers were estimated to disclose their MS at their workplace [29]. Moreover, our findings also showed that manual workers disclosed significantly more than office workers. Managers were the least likely to disclose their diagnosis—however, the level of seniority was unknown. A potential explanation for these occupational differences could be related to the physical work demands being incompatible with their MS. Furthermore, disclosure of the diagnosis in the workplace is observed to differ by most limiting symptom. Accordingly, disclosure is less likely amongst PwMS experiencing “invisible symptoms” like fatigue or cognitive difficulties than physical disabilities [30]. Moreover, receiving work-related adjustments is reliant upon a supportive work environment [15] and often necessitates disclosure. Thus, manual workers might have been compelled to disclose their diagnosis to a greater extent than the other types of occupations to continue working. This is despite this group being younger and having fewer years of disease duration than the other types of occupations. These findings are consistent with a previous study on the disclosure of disabilities amongst young employees suggesting that the timing of disclosure was not only dependent upon the type of disability and level of severity but also on their type of job and industry, and on how comfortable they are in disclosing their condition [31]. It could also be presumed that managers do not need to disclose this information in the same way, as they may be responsible for decisions regarding their work adjustments and work conditions. Furthermore, managers may not need to disclose to the same extent as they also reported higher rates of no-limiting symptoms and less invisible symptoms compared to office workers [29]. In addition, a health selection effect must not be disregarded, as PwMS with better health may lead to a higher status in the labour market [32]. Hence, “occupation may also be affected by health selection, affecting career trajectories, occupational status, and occupational class because unhealthy people are less able to invest energy and time in their occupation and because of labour market discrimination against unhealthy people” (Hoffmann et al., [32], as cited in Kröger, 2015 [33]). Although on average, more than half of the participants responded “always, often or sometime” when asked about having the opportunity to influence their work conditions, managers still had significantly higher positive response rates compared to office and manual workers. Therefore, the clinical profile and occupational characteristics seem to influence disclosure patterns. Likewise, disclosure of diagnosis is often needed to obtain work adjustments.

Overall, the reported adjustments the participants found, or considered would most facilitate their work, were mostly related to special equipment or environment (12.7%), followed by adapted working schedule (8.8%), and customised tasks (4.1%). Office workers had higher rates for adapted schedules when compared to managers, whilst the latter had significantly higher rates of non-response when compared to the rest of occupations, related to their response of “no need” for adjustments at work.

Despite most of the participants reporting limiting symptoms because of their MS, both physical and invisible, adjustments at work were seldom required (20%). Managers reported even fewer adjustments (14%) than office and manual workers (21% and 22%, respectively). These rates are similar to other survey studies exploring workplace adjustments amongst PwMS in the US, where close to 25% of the surveyed PwMS reported having some kind of adjustment at their workplace [17, 34]. Further, these findings both suggest the underuse of adjustment opportunities by people with chronic disabilities as reported by Boot et al.[13]. It is possible that the most commonly offered work adjustments are meant to meet the more visible and “recognisable” needs, associated with a more severe level of MS (e.g. workspace prepared for wheelchairs, adapted technological support for difficulties using upper limbs) and conversely, do not meet the needs for those with “invisible” symptoms (e.g. fatigue, pain, etc.), which might even need a more tailored type of adjustment. Another possibility is that these invisible symptoms are not even recognised as a consequence of the disease and thus, no support is asked for to mitigate their limitation. Regardless, many other factors may be prompting this barrier effect. Dong and Mamboleo, 2022 [19] previously indicated other factors that may hinder PwMS from requesting work accommodations, such as the employers’ lack of knowledge on the rights of people with disabilities or related regulations; the employers’ discrimination and resistance to accommodation requests; the employees’ fear/anxiety to request due to associated stigma; the employees’ lack of knowledge about accommodations/disability/resources, as well as inaccessible workspace. Accordingly, advocating and informing PwMS about the possibilities of workplace adjustments is important, and this needs to be conducted proactively in early MS, as failure to request support may deprive of simple and cost-effective adjustments. The lack of appropriate adjustments limits the potential to promote the work ability of PwMS.

Having any adjustment at work was associated with sociodemographic (women, lower educational level, not being born in Sweden, not living with children, and living outside cities) and clinical (progressive type of MS, higher EDSS scores, and reporting invisible symptoms as the most limiting symptom) characteristics. Strong evidence of MS clinical features in relation to several work-related difficulties PwMS encounter has been found in a previous literature review [2]. High EDSS is established to be associated with reduced work capacity and is a determinant of work-related problems [2]. Further, “invisible” MS symptoms such as fatigue, presence of pain and cognitive dysfunction as well as “visible symptoms” such as mobility, were associated with the likelihood of reducing the amount of work, changing type of work, and leaving work [2]. Moreover, and in accordance with our findings, treatment was not associated with types of adjustments, yet in the literature, it has been shown to improve the work situation amongst PwMS, such as increasing the number of working hours and reducing the length of sick-leave periods [35]. We add the importance of the type of occupation to this discussion on the working life of PwMS. Manual workers were observed to have higher odds to report not having enough support at work than office workers, whilst managers were less likely to have any support at work.

Interestingly, we observed that men had a lower likelihood of having work adjustments compared to women, consistently in all adjusted models. In contrast, Harnett et al. (2014) did not find gender differences regarding employment adjustments for people with disabilities [36]. However, the cited study was not diagnosis-specific but assessed individuals with disabilities, and thus, a sample selection bias could be present, as the data analysed was a systematic collection of individuals who chose to use the Job Adjustments Network and participate in their project [36]. However, one significant finding on gender was that women were less likely than men to find the granted adjustments to be effective. This finding could also indicate that the individual perception of needed support or its efficiency, might not only be subjected to perceived disability but also to other more complex psychological attributes such as the acceptance of the symptoms or the disease. In fact, a study exploring the factors that influence work participation for PwMS indicated that heterogeneity exists regarding the factors that influence employment decisions. Specifically, attributes concerning the impact of work, attitudes in the workplace, and job flexibility appear more significant than those concerning physical workplace adjustments [37]. Although the reason for requesting work adjustments was beyond the scope of this study, future studies are needed to further understand the complex associations between MS disability, progression, and work adjustments (e.g. disease acceptance, drivers for disclosure, perceived efficiency of work adjustments). Moreover, the highly reported “invisible limitations” among PwMS and absence of work adjustments to address these symptoms, require further research on how to create learning opportunities and sensitivity in workplaces to individuals with invisible disabilities.

This study is a first step in providing insight into the choices and experiences of work adjustments amongst more than 3000 PwMS in paid work in Sweden. In addition, participants provided a free-form response to the work adjustment that they considered most important, without the constraint of fixed answers. A disadvantage was the lack of response which could partly be explained by how the question was framed (following if they had any work adjustments) or how participants interpret it (e.g. they might consider it irrelevant). Further, the fact of having support at work does not necessarily mean that they also consider it useful. Therefore, the lack of response might also be reflecting this. It is also possible that those who do not have the opportunity to request work adjustments have already left their workplace, as the clinical profile depicted most of the participants in the early phase of the disease. Yet, the socio-demographic profile of the participants reflected other MS cohorts and similar sex distribution in the labour market to the wider trends in Sweden [7, 8, 11, 38]. Another limitation to our study was the lack of information on the PwMS who did not respond to the survey, which could have provided more insights on the topic and minimised the bias on our results by assigning weights to the different responses. Further, a more granular description by sectors or types of jobs within each type of occupation could bring further knowledge to the research area. Nonetheless, to the best of our knowledge, this is the first study to explore if PwMS report support and of what type in relation to different types of occupations, reported MS limitations, disclosure status, and other socio-demographic and clinical characteristics. Nevertheless, more research regarding the nuances and fine-tuning of specific work adjustments (e.g. Why do not PwMS request a work adjustment if they disclosed MS at work and acknowledged having a limiting symptom?) will necessitate in-depth interviews to understand and suggest solutions to further assist PwMS in remaining in work.

## Conclusion

Work adjustments have the potential to facilitate opportunities for work retention and work development for people with a chronic disease like MS. Our findings indicate that an advanced clinical profile of MS together with disclosure at work and perceived MS limitations are associated with having work adjustments amongst employed PwMS. Nevertheless, the type of occupation may have a modulating effect on the request for these adjustments as well as the opportunity to influence work-related conditions. These differences are mainly observed amongst those with managerial occupations who might have more flexibility to accommodate their limitations to their work demands as they often have the power and decision-making authority for themselves. Moreover, further considerations are needed towards educating employees and employers regarding invisible symptoms which may not be sufficiently recognised as limiting their work capacity and hence, not considered as qualifying for requesting appropriate work adjustments.

## Supplementary Information

Below is the link to the electronic supplementary material.

Supplementary file1 (DOCX 29 KB)

## Data Availability

The data used in the study cannot be made public in accordance with the General Data Protection Regulation, the Swedish Data Protection Act, the Swedish Ethical Review Act, and the Swedish Public Access to Information and Secrecy Act. This type of sensitive data can only be made available to researchers after legal review, if meeting the criteria to access this type of sensitive and confidential data. Readers may contact Associate Professor Emilie Friberg (emilie.friberg@ki.se) for further information regarding this data.
