# Supplementary material for: Work Adjustments by Types of Occupations Amongst People with Multiple Sclerosis: A Survey Study
Source: J Occup Rehabil. 2023 Nov 3;34(2):461–71. doi: 10.1007/s10926-023-10142-2 (PMC11180149; doi:10.1007/s10926-023-10142-2)
Supplement: Supplementary file 1 — Supplementary file1 (DOCX 29 KB) [file 10926_2023_10142_MOESM1_ESM.docx]

# SUPPLEMENTARY MATERIAL

**Supplementary Table 1.** Absolute numbers and proportions of the survey responses reporting work adjustments by the type of adjustment they consider to be or would be the most important to facilitate their work (n=3299)

|  | **Type of work adjustment** | | | | |
| --- | --- | --- | --- | --- | --- |
|  | N/A | Special equipment/  environment | Customised tasks | Adapted working schedule | Other support |
|  | (n=2390) | (n=421) | (n=137) | (n=293) | (n=58) |
|  | n(%) | n(%) | n(%) | n(%) | n(%) |
| ***Have you received any adjustments that helps you with your work?*** | | | | | |
| Yes ^a, b^ | 45(1.9) | 230(54.6) | 57(41.6) | 144(49.1) | 25(43.1) |
| Yes, but not enough ^a^ | 17(0.7) | 74(17.6) | 26(19.0) | 42(13.3) | 13(22.4) |
| No, but I do need | 27(1.1) | 117(27.8) | 54(39.4) | 107(36.5) | 20(34.5) |
| No, not needed | 2301(96.3) | 0(0) | 0(0) | 0(0) | 0(0) |
| **Total (n=2399)** | 2390(72.4) | 421(12.8) | 137(4.2) | 293(8.9) | 58(1.8) |

Chi2 tests were performed to compare proportions. Results are based on two-sided tests and adjusted for all pairwise comparisons using the Benjamini-Hochberg procedure. Missing cases were excluded for analyses purposes.

^a^ Proportional difference between N/A and all other types of adjustments (p< 0.05).

^b^ Proportional difference between Special equipment/environment and Adapted task (p< 0.05).

**Supplementary Table 2**: Multinomial logistic regression analyses of the associations of having any work adjustments and sociodemographic or clinical characteristics and reported Survey questions (crude model)

|  | **Crude model** | | |
| --- | --- | --- | --- |
|  | **OR [95% CI]** | | |
|  | “Yes” | “Yes, but not enough” | “No, but I do need” |
| **Sex** (ref.= Women) |  |  |  |
| Men | **0.62 [0.49 - 0.77]** | **0.56 [0.38 - 0.82]** | **0.49 [0.36 - 0.65]** |
| **Age, years** (ref.= 40-49) |  |  |  |
| 20-29 | **0.47 [0.31 - 0.72]** | 0.64 [0.34 - 1.22] | 1.34 [0.92 - 1.96] |
| 30-39 | **0.65 [0.52 - 0.81]** | 0.88 [0.62 - 1.24] | 1.03 [0.80 - 1.33] |
| 50+ | 1.32 [0.91 - 1.92] | 1.24 [0.66 - 2.33] | **0.48 [0.23 - 0.99]** |
| **Educational level** (ref.= University) |  |  |  |
| Non university | **1.54 [1.26 - 1.87]** | 1.31 [0.95 - 1.80] | 1.21 [0.95 - 1.54] |
| **Country of birth** (ref.= Sweden) |  |  |  |
| Not Sweden | 1.14 [0.83 - 1.57] | 1.57 [0.99 - 2.46] | **1.47 [1.04 - 2.09]** |
| **Living with children** (ref.=yes) |  |  |  |
| No | 1.20 [0.99 - 1.46] | 1.25 [0.91 - 1.70] | 1.19 [0.94 - 1.51] |
| **Civil status** (ref.=Married/cohabitant) |  |  |  |
| Single/separated/widowed | 0.87 [0.72 - 1.05] | 0.97 [0.71 - 1.32] | 1.13 [0.89 - 1.43] |
| **Type of living area** (ref.= Cities) |  |  |  |
| Towns and suburbs | **1.40 [1.13 - 1.74]** | 0.98 [0.70 - 1.39] | 1.04 [0.81 - 1.34] |
| Rural areas | **1.77 [1.36 - 2.31]** | 1.24 [0.81 - 1.90] | 0.89 [0.63 - 1.27] |
| **Occupations** (ref.= Office) |  |  |  |
| Managers | **0.51 [0.37 - 0.72]** | 0.81 [0.50 - 1.30] | **0.62 [0.42 - 0.91]** |
| Manual | 0.94 [0.64 - 1.39] | 1.40 [0.81 - 2.40] | 1.05 [0.67 - 1.65] |
| **MS type** (ref.= RRMS) |  |  |  |
| Progressive MS (PPMS & SPMS] | 4.84 [3.41 - 6.89] | 4.66 [2.81 - 7.74] | 1.32 [0.72 - 2.41] |
| **MS severity** (ref.= EDSS=1-2.5) |  |  |  |
| EDSS=0 | **0.38 [0.28 - 0.51]** | **0.25 [0.13 - 0.46]** | **0.51 [0.36 - 0.70]** |
| EDSS=3-9.5 | **4.41 [3.35 - 5.79]** | **4.81 [3.24 - 7.15]** | **2.33 [1.62 - 3.34]** |
| **Ongoing MS treatment** (ref.= yes) |  |  |  |
| No | 1.21 [0.87 - 1.69] | 0.99 [0.56 - 1.74] | 0.81 [0.51 - 1.28] |
| **Years since MS diagnosis** (ref.= 0-4) |  |  |  |
| 5-9 | 1.12 [0.86 - 1.47] | 0.72 [0.48 - 1.09] | 0.85 [0.63 - 1.14] |
| 10-14 | 1.21 [0.91 - 1.61] | **0.61 [0.38 - 0.98]** | 0.73 [0.53 - 1.01] |
| 15 | **1.43 [1.07 - 1.91]** | 0.80 [0.50 - 1.26] | **0.58 [0.40 - 0.84]** |
| **Most limiting symptom** (ref.= Visible symptoms) |  |  |  |
| Invisible symptom | 1.09 [0.85 - 1.40] | **1.68 [1.07 - 2.63]** | **1.60 [1.15 - 2.23]** |
| No symptoms | **0.12 [0.07 - 0.22]** | **0.11 [0.03 - 0.36]** | **0.05 [0.02 - 0.18]** |
| **Disclosed MS diagnosis to their boss** (ref.=Yes) |  |  |  |
| No | **0.10 [0.06 - 0.16]** | **0.23 [0.13 - 0.41]** | **0.62 [0.46 - 0.84]** |

Reference group (outcome): "No, not needed"

Bold numbers indicate a p <0.05, two-sided test.

*Abbreviations:* OR, Odds Ratio; CI, Confidence intervals; ref.=references; RRMS, Relapsing-remitting MS; PPMS, Primary progressive MS; SPMS, secondary progressive MS; EDSS, Expanded Disability Status Scale
